# Signaling in the phytomicrobiome: breadth and potential

**DOI:** 10.3389/fpls.2015.00709

**Published:** 2015-09-09

**Authors:** Donald L. Smith, Sowmyalakshmi Subramanian, John R. Lamont, Margaret Bywater-Ekegärd

**Affiliations:** ^1^Plant Science Department, McGill University/Macdonald Campus, Sainte-Anne-de-Bellevue, QCCanada; ^2^Inocucor Technologies Inc., Montréal, QCCanada

**Keywords:** molecular signals, plant growth promoting rhizobacteria, phytomicrobiome, holobiont, crop

## Abstract

Higher plants have evolved intimate, complex, subtle, and relatively constant relationships with a suite of microbes, the phytomicrobiome. Over the last few decades we have learned that plants and microbes can use molecular signals to communicate. This is well-established for the legume-rhizobia nitrogen-fixing symbiosis, and reasonably elucidated for mycorrhizal associations. Bacteria within the phytomircobiome communicate among themselves through quorum sensing and other mechanisms. Plants also detect materials produced by potential pathogens and activate pathogen-response systems. This intercommunication dictates aspects of plant development, architecture, and productivity. Understanding this signaling via biochemical, genomics, proteomics, and metabolomic studies has added valuable knowledge regarding development of effective, low-cost, eco-friendly crop inputs that reduce fossil fuel intense inputs. This knowledge underpins phytomicrobiome engineering: manipulating the beneficial consortia that manufacture signals/products that improve the ability of the plant-phytomicrobiome community to deal with various soil and climatic conditions, leading to enhanced overall crop plant productivity.

## Background

Most energy in the terrestrial biosphere enters it through photosynthesis (Imhoff et al., 2004) carried out by plant leaves (Luo et al., 2006). Non-photosynthetic organisms with reliable access to plant energy are in an advantaged situation. Under natural conditions higher plants are always associated with a complex and relatively constant microflora (Rout and Southworth, 2013; Turner et al., 2013a). Terrestrial plants release ∼20% of photosynthetically fixed carbon as root exudates, resulting in an energy rich rhizosphere (Kuzyakov and Domanski, 2000), and a rich, generally compositionally consistent phytomicrobiome (Bulgarelli et al., 2012; Hirsch and Mauchline, 2012; Lundberg et al., 2012). These exudates vary among species, specific genotypes within species, stages of plant development and growing conditions, and influence the composition of the rhizomicrobiome (Bascom-Slack et al., 2012; Marasco et al., 2012; Badri et al., 2013a,b; Turner et al., 2013a,b; Chaparro et al., 2014).

Phytomicrobiome associations are analogous to the animal microbiome (Koenig et al., 2011); microbiome diversity, stability, and resilience play a large role in human health and disease (Cho and Blaser, 2012). Plants have likely had associated microbes since they colonized the land, almost half a billion years ago; roots of the first terrestrial plants were almost certainly less sophisticated than those that followed, making these early plants more in need of microbial assistance (Knack et al., 2015). Fossil endomycorrhizal associations occur in the early Devonian period, demonstrating association of plant roots with fungal elements of the rhizomicrobiome (Taylor, 1995; Bonfante and Genre, 2008; Porras-Alfaro and Bayman, 2011). Mycorrhizal relationships are sophisticated and their presence >400 million years ago indicates that the phytomicrobiome had already been developing for some time; it seems likely that bacterial associations have been present for at least as long. As plants adapted to and spread through diverse terrestrial environments, evolving to grow under a range of conditions, it is probable that their associations with microbes also evolved. This community of microbes is the phytomicrobiome (Smith and Zhou, 2014), with its root associated (Hirsch and Mauchline, 2012; Lundberg et al., 2012; rhizomicrobiome), above ground associated (Rastogi et al., 2012, 2013; Badri et al., 2013b; Kembel et al., 2014; phyllomicrobiome) and interior (Berg et al., 2014; endosphere) components. Even “lower plants” such as *Sphagnum* sp. have complex phytomicrobiomes, including highly specific associations with diazotrophs (Bragina et al., 2013).

Hence, a plant growing in nature is not a single organism; it is a community: a holobiont (Hartmann et al., 2014). While a plant growing in isolation can be very useful for research purposes, it is an anomaly. Like the human microbiome, the phytomicrobiome constitutes an underappreciated biological aspect (physiology, genome, metabolome, etc.) of plants. Plants and their associated phytomicrobiome affect each other in various and subtle ways (Berendsen et al., 2012); a field-grown plant is a meta-organism (Berg et al., 2013), having a persistent and regulated relationship with its phytomicrobiome. The composition of the phytomicrobiome is regulated by numerous biotic and abiotic factors including the complex matrix of plant–microbe and microbe–microbe communications. This communication is carried out through the release of signaling compounds, the forms and functions of which are currently being elucidated. This new understanding can be exploited to: (1) develop new approaches to crop growth promotion, (2) optimize related fermentation and formulation processess, and (3) develop novel and more consistent biocontrol mechanisms for field crops (East, 2013).

## The Phytomicrobiome and Plant Growth

There has been an upsurge in phytomicrobiome publications; this community of microbes is now seen as key to the growth and health of plants (Schmidt et al., 2014); there is still a great deal to be learned about the composition and nature of interactions among members of this community, and its interactions with the host plant.

Microbes associate with the phyllosphere (as both epi- and endophytes, of leaves and stems), rhizosphere and reproductive structures such as flowers, fruits and seeds. In grape, *Pseudomonas* and *Bacillus* spp. colonize the epidermis and xylem of the ovary and ovules, while *Bacillus* spp. colonize berries and seed cell walls (Lugtenberg and Kamilova, 2009; Compant et al., 2010a,b). Nitrogen-fixing plant growth promoting rhizobacteria (PGPR; Loiret et al., 2004; Quecine et al., 2012; e.g., *Acetobacter diazotrophicus, Pantoea agglomerans* 33.1) associate with plant roots (Pisa et al., 2011), and stems of sugarcane (Velázquez et al., 2008), residing in the apoplast in a low-nitrogen, high-sucrose environment (Dong et al., 1994). Other nitrogen-fixing bacteria (*Azotobacter*, *Enterobacter*, *Bacillus*, *Klebsiella*, *Azospirillum*, *Herbaspirillum*, *Gluconacetobacter*, *Burkholderia, Azoarcus*) are found in grasses such as rice and maize (Von Bulow and Dobereiner, 1975; James, 2000; Baldani et al., 2002; Boddey et al., 2003; Santi et al., 2013). Phyllomicrobiome communities influence plant development and ecosystem function, while the host controls aspects of phytomicrobiome composition and function. Environmental factors are known to alter biosynthesis of many metabolites within plants; specific members of the rhizomicrobiome also alter plant development, growth, and composition. Treatment of leaves with specific phyllomicrobiome components suppresses feeding by insect larvae (Badri et al., 2013b). The distribution and community composition of microbes in the phyllosphere is thought to be somewhat random, whereas plants create niches in the rhizosphere and endosphere to accommodate specific microbial communities (Lebeis, 2015).

The rhizomicrobiome is comprised of diverse root endophytes (Gaiero et al., 2013), some of which are PGPRs. Compositionally the rhizomicrobiome is dynamic in time and space, in response to environmental conditions, the presence of other soil organisms, soil physical conditions, plant species and genotype and interactions between a specific microbe and a specific plant type. The best characterized microbes in the rhizomicrobiome are the PGPR. These include bacteria in the soil near plant roots, on the surface of plant root systems, in spaces between root cells or inside specialized cells of root nodules; they stimulate plant growth through a wide range of mechanisms (Gray and Smith, 2005; Mabood et al., 2014), such as: (1) nutrient solubilization (particularly phosphorus – Boddey et al., 2003; Kennedy et al., 2004; Trabelsi and Mhamdi, 2013), (2) production of metal chelating siderophores, (3) nitrogen fixation (Vessey, 2003; Bhattacharyya and Jha, 2012; Drogue et al., 2012), (4) production of phytohormones, (5) production of 1-aminocyclopropane-1-carboxylate deaminase, (6) production of volatile organic compounds, (7) induction of systemic resistance [induced systemic resistance (ISR) and systemic required resistance (SAR) – Jung et al., 2008b, 2011], and (8) suppression of disease through antibiosis (Bhattacharyya and Jha, 2012; Spence et al., 2014). It has also been shown that “signal” compounds produced by bacteria in the phytomicrobiome stimulate plant growth (Prithiviraj et al., 2003; Mabood et al., 2006a; Lee et al., 2009), particularly in the presence of abiotic stress (Wang et al., 2012; Subramanian, 2014; Prudent et al., 2015). In the broadest sense PGPR include legume-nodulating rhizobia. PGPR reside outside plant cells (extracellular – ePGPR) or, like rhizobia, live inside them (intracellular – iPGPR; Gray and Smith, 2005). Application of PGPR to crops, except for rhizobia, has met with mixed results in the field, causing increased growth sometimes and not others (Nelson, 2004). Elements of the phytomicrobiome also assist plants in dealing with abiotic stress. The *Arabidopsis* phytomicrobiome, for instance, can sense drought stress and help the plant maintain productivity (Zolla et al., 2013). Further, mycorrhizal associations enhance crop salinity tolerance (Porcel et al., 2012; Ruiz-Lozano et al., 2012). At a time when we are looking to crop plants to provide biofuels and other bioproducts while still feeding the world’s growing population, against a background of climate change, understanding and developing technologies that can increase overall plant productivity is imperative (Ragauskas et al., 2006; Babalola, 2010; Dutta and Podile, 2010; Beneduzi et al., 2012; Orrell and Bennett, 2013).

Newer deployments of PGPR and/or arbuscular mycorrhizal fungi (AMF) consortia that promote crop productivity by mimicking, or partially reconstructing, the phytomicrobiome are being developed. Application of a PGPR consortium (*Bacillus amyloliquefaciens* IN937a, *Bacillus pumilus* T4, AMF *Glomus intraradices*) to greenhouse tomato resulted in full yield with 30% less fertilizer (Adesemoye et al., 2009). Co-inoculation of *B. japonicum* 532C, RCR3407 and *B. subtilis* MIB600 increased biomass for two soybean cultivars (Atieno et al., 2012). Co-inoculation of *B. japonicum* E109 and *Bacillus amyloliquefaciens* LL2012 improved soybean nodulation efficiency. Phytohormone production by *B. amyloliquefaciens* LL2012 improved nodulation efficiency for *B. japonicum* E109 (Masciarelli et al., 2014). A consortium of *B. megaterium*, *Enterobacter* sp., *B. thuringiensis* and *Bacillus* sp., plus composted sugar beet residue, on *Lavandula dentata* L. helped restore soils by increasing phosphorus availability, soil nitrogen fixation and foliar NPK content (Mengual et al., 2014).

## Signaling in the Phytomicrobiome

The complex community formed by the plant and its phytomicrobiome is carefully orchestrated; there is signal exchange among the various microbes involved, and also between the host plant and the microbe community (Engelmoer et al., 2014). These signals regulate aspects of each other’s activities and the community overall. Microbial chemical signals can help plants initiate immune responses to harmful pathogens or allow the entry of beneficial endophytes (Hartmann et al., 2014). Microbe associated molecular patterns (MAMPs) play a key role in plant immune response and antibiotic secretion in microbes. Plant associated *Bacillus* strains have been shown to down-regulate MAMP-regulated immune response including antibiotic secretion in the presence of plant root exudates to better facilitate root infection (Lakshmanan et al., 2012). Bacteria can also interfere with signaling between plants and other microbial strains. LCOs are similar in structure to chitin and can be cleaved by bacterially produced chitinases, thus interfering with plant microbe symbioses (Jung et al., 2008a). Other aspects plant–microbe symbiosis follow pathways similar to pathogen infection (Barea, 2015).

Signaling compounds produced by plants include a variety of root exudates such as primary metabolites (carbohydrates, proteins, organic acids, etc.) and secondary metabolites (flavonoids, phenol, phytohormones, etc.). Plants often excrete more of these signaling compounds in response to stress. PGPR-to-plant signaling compounds include phytohormones, acyl homoserine lactones, phenols and peptides and can also act as microbe to microbe signals (Barea, 2015). Root exudates signal and recruit specific microbial communities. Secretion of malic acid in *Arabidopsis thaliana* in response to foliage pathogen attack stimulates the formation of beneficial biofilms in the rhizosphere (Rudrappa et al., 2008).

That plants and microbes use signal compounds to communicate during establishment of beneficial plant-microbe interactions (Desbrosses and Stougaard, 2011), is well-described for the legume-rhizobia nitrogen fixing symbiosis (Oldroyd et al., 2010; Giles et al., 2011; Oldroyd, 2013), and somewhat elucidated for mycorrhizal associations (Gough and Cullimore, 2011). In the legume-rhizobia relationship the plant releases flavonoid signals to rhizobia (Hassan and Mathesius, 2012) or, in some cases, jasmonate signals (Mabood et al., 2006a,b; Mabood et al., 2014), followed by rhizobial production of lipo-chitooligosaccharides (LCOs) as return signals (Oldroyd, 2013). The LCOs are bound by LysM receptors, which have kinase activity (Antolin-Llovera et al., 2012), changing root hormone profile (Zamioudis et al., 2013) and triggering development of root nodules. Plants also communicate with, or otherwise influence the phytomicrobiome, affecting its composition and structure (Delaux et al., 2012; Badri et al., 2013a; Bálint et al., 2013; Peiffer et al., 2013; Turner et al., 2013b; Venkateshwaran et al., 2013; Chaparro et al., 2014; Evangelisti et al., 2014). Bacteria also communicate among themselves (Cretoiu et al., 2013); quorum sensing via *N*-acyl homoserine lactone (Teplitski et al., 2000) is well-characterized, and there are likely other, as of yet unknown, mechanisms (Lv et al., 2013). Quorum sensing signals can trigger immune responses and changes in hormone profiles in plants, leading to growth responses (Hartmann and Schikora, 2012). Quorum sensing in the phytomicrobiome will be the subject of an upcoming Frontiers in Plant Science theme volume (Plant responses to bacterial quorum sensing signal molecules, topic editors Schikora A, Hartmann A, and Munchen HZ). This sort of signaling almost certainly occurs in the phytomicrobiome. Plants also detect materials produced by potential pathogens and respond by activating response systems (Tena et al., 2011). Phytomicrobiome intercommunication in the rhizosphere dictates aspects of above-ground plant architecture and above-ground symbiotic/pathogenic microbial communities (Segonzac and Zipfel, 2011; Tena et al., 2011). Similarly, pathogen or herbivore attacks above ground can effect microbial community composition in the rhizosphere. Above ground injury has been shown to stimulate the production of signaling compounds in plant roots (Lakshmanan et al., 2012). Greater photosynthetic rates under elevated CO_2_ conditions have been shown to change microbial community composition in the rhizosphere (Berlec, 2012; He et al., 2012). Understanding plant responses to microbial signals via proteomics (Elmore et al., 2012; Nguyen et al., 2012; Rose et al., 2012) and metabolomics (Watrous et al., 2012; Zhang et al., 2012) studies has added valuable knowledge toward developing effective low-cost and eco-friendly practices to reduce fossil-fuel dependent crop inputs, leading to interest in phytomicrobiomes engineered to enhanced plant growth under variable soil and climatic conditions, improving global crop productivity.

Surprisingly, LCOs are also able to stimulate plant growth directly (Souleimanov et al., 2002; Prithiviraj et al., 2003; Almaraz et al., 2007; Khan et al., 2008; Wang et al., 2012); confirmed by Oláh et al. (2005) for root growth in *Medicago truncatula*, Chen et al. (2007) for accelerated flowering (a typical response to stress) and increased yield in tomato, and stimulation of early somatic embryo development in Norway spruce (Dyachok et al., 2002). Enhanced germination and seedling growth, along with the mitogenic nature of LCOs, suggest accelerated meristem activity. Products based on LCOs are now used to treat seed sown into several 10s of million ha of crop land each year, largely corn and soybean. A similar jasmonate product is now available. The effects of LCOs are much greater when stress (salt, drought, cold) is present than under optimum conditions (Smith, 2009, 2010; Subramanian et al., 2009, 2010, 2011; Schwinghamer et al., 2014; Subramanian, 2014; Prudent et al., 2015). Thuricin 17, a bacteriocin produced by *Bacillus thuringiensis* NEB17 isolated from soybean roots, improves plant growth and resilience to stress (Schwinghamer et al., 2014; Subramanian, 2014). Inhibition of legume nodulation, and of overall plant growth, by stressful conditions can be overcome by LCOs (nodulation – Zhang and Smith, 1995, 2002; plant growth – Schwinghamer et al., 2014; Prudent et al., 2015); Estévez et al. (2009) showed that at least one rhizobial strain produce different LCOs when grown under salt stress, and that salt stress itself can induce the *nod* genes of this strain (Guasch-Vidal et al., 2013).

## Future Directions

We now understand that the phytomicrobiome is a complex, structured and dynamic community with a relatively constant set of potential members, whose relative abundances can shift within plant species and their genotypes, and in response to both abiotic conditions and plant development, leading to dynamism in the communications among the microbial community and the host plants. Methods, such as high throughput genotyping, are allowing us to determine the taxonomic diversity of the phytomicrobiome (Hirsch and Mauchline, 2012; Peiffer et al., 2013; Turner et al., 2013b). A better understanding of plant signaling may also become a tool for investigating community composition of the phytomicrobiome. Root exudates play an important role in the formation of microbial communities in the rhizosphere and can be useful in predicting community compositions (Berg et al., 2014). Correlations between phytomicrobiome bacterial diversity and host growth, mortality, and function suggest that incorporating information on plant–microbe associations will improve our ability to understand plant functional biogeography and drivers of variation in plant and ecosystem function (Kembel et al., 2014). It has even been suggested that beneficial effects of the phytomicrobiome could be enhanced through plant breeding, developing genotypes that encourage best membership in the phytomicrobiome (Bakker et al., 2012). More effective methods to study plant MAMP receptors are being developed (Wittulsky et al., 2014) and could lead to ways to engineer plant recognition receptors.

Novel methods of manipulating signaling in the phytomicrobiome could lead to crop production practices that are less reliant on non-renewable resources and crops more resilient in the face of stresses (Marasco et al., 2012), most crucially, those associated with climate change. Plant stress response seems to play an important role in the release of signaling compounds in the rhizosphere but the specifics of this interaction are still unclear. A better understanding of the relationship between environmental plant stress and signaling could help in developing technologies that utilize plant signaling in crop stress alleviation (Barea, 2015).

Recent developments have shown that temperature (Schwinghamer et al., 2014) and water stress (Prudent et al., 2015) can influence plant microbe communication. Environmental factors likely play an important and underdescribed role in signaling in the phytomicrobiome. Variable environmental factors may account for some of the inconsistency observed in field trials of microbial products that previously yielded favorable results in laboratory conditions. A more complete understanding of how plant–microbe communication is influenced by environmental factors will likely be useful in achieving more consistent results with agricultural microbial products.

Despite being at an early stage in understanding these communities, it is clear that there is considerable potential for application of coordinated microbial consortia to crop agriculture and, thus, to enhancing global food security. While advances in methods and technologies in microbiology used to investigate non-culturable microbial strains have led to a stronger focus on a community level approach to plant–microbe interaction research (Berlec, 2012; Rastogi et al., 2013), isolated, culturable microbial strains are still required for most plant–microbe signaling research, particularly if the research is aimed at developing commercial microbial products. Culturable strains are needed both to produce a consistent product and to verify growth promotion through plant growth trials. There are clear opportunities for development of products for more sustainable agronomic production systems (Kloepper et al., 2004; De-la-Peña and Loyola-Vargas, 2014). A range of PGPR have been identified, and even developed into products utilized in crop production. Signaling compounds that directly stimulate plant growth or improve stress tolerance have great potential because they can be produced by microbes in a controlled bioreactor rather than in variable field conditions as with inoculants. The global market for biostimulants has been projected to reach $2.241 million by 2018 and to have a compounded annual growth rate of 12.5% from 2013 to 2018 (Calvo et al., 2014). Products based on multispecies consortia may address consistency in performance observed in single species inoculants. Industry is working to harness the knowledge surrounding the phytomicrobiome, to quickly bring sustainable, consortia-based products to production agriculture.

## Conflict of Interest Statement

The fourth author is Executive Vice President of Technology and Innovation at Inocucor Technologies, a company that manufactures and sells microbial consortia for plants; the first author conducts research in collaboration with this company, where the research is funded through a Canadian Federal Government program (Mitacs) which levers industrial funding.

## References

[B1] Adesemoye A. O. Torbert H. A. Kloepper J. W. (2009). Plant growth promoting rhizobacteria allow reduced application rates of chemical fertilizers. *Microb. Ecol.* 58 921–929. 10.1007/s00248-009-9531-y 19466478

[B2] Almaraz J. Zhou X. Smith D. L. (2007). Gas exchange characteristics and dry matter accumulation of soybean treated with Nod factors. *J. Plant. Phys.* 164 1391–1393. 10.1016/j.jplph.2006.12.007 17485139

[B3] Antolin-Llovera M. A. Ried M. K. Binder A. Parniske M. (2012). Receptor kinase signaling pathways in plant-microbe interactions. *Annu. Rev. Phytopathol.* 50 451–473. 10.1146/annurev-phyto-081211-173002 22920561

[B4] Atieno M. Herrmann L. Okalebo R. Lesueur D. (2012). Efficiency of different formulations of Bradyrhizobium japonicum and effect of co-inoculation of *Bacillus subtilis* with two different strains of *Bradyrhizobium japonicum*. *World. J. Microbol. Biotechnol.* 28 2541–2550. 10.1007/s11274-012-1062-x 22806160

[B5] Babalola O. O. (2010). Beneficial bacteria of agricultural importance. *Biotechnol. Lett.* 32 1559–1570. 10.1007/s10529-010-0347-0 20635120

[B6] Badri D. V. Chaparro J. M. Zhang R. Shen Q. Vivanco J. M. (2013a). Application of natural blends of phytochemicals derived from the root exudates of *Arabidopsis* to the soil reveal that phenolic-related compounds predominantly modulate the soil microbiome. *J.Biol. Chem.* 288 4502–4512. 10.1074/jbc.M112.433300 23293028 PMC3576057

[B7] Badri D. V. Zolla G. Bakker M. G. Manter D. K. Vivanco J. M. (2013b). Potential impact of soil microbiomes on the leaf metabolome and on herbivore feeding behavior. *New Phytol.* 198 264–273. 10.1111/nph.12124 23347044

[B8] Bakker M. G. Manter D. K. Sheflin A. M. Weir T. L. Vivanco J. M. (2012). Harnessing the rhizosphere microbiome through plant breeding and agricultural management. *Plant Soil* 360 1–13. 10.1007/s11104-012-1361-x

[B9] Baldani J. I. Reis V. M. Baldani V. L. D. Dobereiner J. (2002). A brief story of nitrogen fixation in sugarcane – reasons for success in Brazil. *Funct. Plant Biol.* 29 417–423. 10.1071/PP01083 32689486

[B10] Bálint M. Tiffin P. Hallström B. O’Hara R. B. Olson M. S. Fankhauser J. D. (2013). Host genotype shapes the foliar fungal microbiome of Balsam Poplar (*Populus balsamifera*). *PLoS ONE* 8:e53987 10.1371/journal.pone.0053987 PMC3543377 23326555

[B11] Barea J. M. (2015). Future challenges and perspectives for applying microbial biotechnology in sustainable agriculture based on a better understanding of plant-microbe interactions. *J. Soil Sci. Plant Nutr.* 15 261–282. 10.4067/S0718-95162015005000021

[B12] Bascom-Slack C. A. Arnold A. E. Strobel S. A. (2012). Student-directed discovery of the plant microbiome and its products. *Science* 338 485–486. 10.1126/science.1215227 23112324

[B13] Beneduzi A. Ambrosini A. Passaglia L. M. P. (2012). Plant growth-promoting rhizobacteria (PGPR): their potential as antagonists and biocontrol agents. *Genet. Mol. Biol.* 35 1044–1051. 10.1590/S1415-47572012000600020 23411488 PMC3571425

[B14] Berendsen R. L. Pieterse C. M. J. Bakker P. A. H. M. (2012). The rhizosphere microbiome and plant health. *Trends Plant Sci.* 8 478–486. 10.1016/j.tplants.2012.04.001 22564542

[B15] Berg G. Grube M. Schloter Kronelia S. (2014). Unraveling the plant microbe: looking back and future perspectives. *Front. Microbiol.* 5:148 10.3389/fmicb.2014.00148 PMC4045152 24926286

[B16] Berg G. Zachow C. Müller H. Philipps J. Tilcher R. (2013). Next-generation bio-products sowing the seeds of success for sustainable agriculture. *Agronomy* 3 648–656. 10.3390/agronomy3040648

[B17] Berlec A. (2012). Novel techniques and findings in the study of plant microbiota: search for plant probiotics. *Plant Sci.* 19 96–102. 10.1016/j.plantsci.2012.05.010 22794922

[B18] Bhattacharyya P. N. Jha D. K. (2012). Plant growth-promoting rhizobacteria (PGPR): emergence in agriculture. *World J. Microbiol. Biotechnol.* 28 1327–1350. 10.1007/s11274-011-0979-9 22805914

[B19] Boddey R. M. Urquiaga S. Alves B. J. R. Reis V. (2003). Endophytic nitrogen fixation in sugarcane: present knowledge and future applications. *Plant Soil* 252 139–149. 10.1023/A:1024152126541

[B20] Bonfante P. Genre A. (2008). Plants and arbuscular mycorrhizal fungi: an evolutionary-developmental perspective. *Trends Plant Sci.* 9 402–498. 10.1016/j.tplants.2008.07.001 18701339

[B21] Bragina A. Berg C. Müller H. Moser D. Berg G. (2013). Insights into functional bacterial diversity and its effects on Alpine bog ecosystem functioning. *Sci. Rep.* 3 1955 10.1038/srep01955 PMC6504810 23739741

[B22] Bulgarelli D. Rott M. Schlaeppi K. van Themaat E. V. L. Ahmadinejad N. Assenza F. (2012). Revealing structure and assembly cues for *Arabidopsis* root-inhibitiing bacterial microbiota. *Nature* 488 91–95. 10.1038/nature11336 22859207

[B23] Calvo P. Nelson L. Kloepper J. W. (2014). Agricultural uses of plant biostimulants. *Plant Soil* 383 3–41. 10.1007/s11104-014-2131-8

[B24] Chaparro J. M. Badri V. D. Vivanco M. J. (2014). Rhizosphere microbiome assemblage is affected by plant development. *ISME J.* 8 790–803. 10.1038/ismej.2013.196 24196324 PMC3960538

[B25] Chen C. McIver J. Yang Y. Bai Y. Schultz B. McIver A. (2007). Foliar application of lipo-chitooligosaccharides (Nod factors) to tomato (*Lycopersicon esculentum*) enhances flowering and fruit production. *Can. J. Plant Sci.* 87 365–372. 10.4141/P06-164

[B26] Cho I. Blaser M. J. (2012). The human microbiome: at the interface of health and disease. *Nat. Rev. Genet.* 13 260–270. 10.1038/nrg3182 22411464 PMC3418802

[B27] Compant S. Clement C. Sessitsch A. (2010a). Plant growth-promoting bacteria in the rhizo- and endosphere of plants: their role, colonization, mechanisms involved and prospects for utilization. *Soil Biol. Biochem.* 42 669–678. 10.1016/j.soilbio.2009.11.024

[B28] Compant S. van der Heijden M. G. A. Sessitsch A. (2010b). Climate change effects on beneficial plant-microorganism interactions. *FEMS Microbiol. Ecol.* 73 197–214. 10.1111/j.1574-6941.2010.00900.x 20528987

[B29] Cretoiu M. S. Korthals G. W. Visser J. H. M. van Elsas J. D. (2013). Chitin amendment increases soil suppressiveness toward plant pathogens and modulates the actinobacterial and oxalobacteraceal communities in an experimental agricultural field. *Appl. Evniron. Microbiol.* 17 5291–5301. 10.1128/AEM.01361-13 PMC3753968 23811512

[B30] Delaux P.-M. Xie X. Timme R. E. Puech-Pages V. Dunand C. Lecompte E. (2012). Origin of strigolactones in the green lineage. *New Phytologist* 195 857–871. 10.1111/j.1469-8137.2012.04209.x 22738134

[B31] De-la-Peña C. Loyola-Vargas V. M. (2014). Biotic interactions in the rhizosphere: A diverse cooperative enterprise for plant productivity. *Plant Physiol.* 166 701–719. 10.1104/pp.114.241810 25118253 PMC4213099

[B32] Desbrosses G. J. Stougaard J. (2011). Root nodulation: A paradigm for how plant-microbe symbiosis influences host developmental pathways. *Cell Host Microbe* 10 348–358. 10.1016/j.chom.2011.09.005 22018235

[B33] Dong Z. Canny M. J. McCully M. E. Roboredo M. R. Cabadilla C. F. Ortega E. (1994). A nitrogen-fixing endophyte of sugarcane stems’. A new role for the apoplast. *Plant Physiol.* 105 1139–1147. 12232271 10.1104/pp.105.4.1139 PMC159442

[B34] Drogue B. Doré H. Borland S. Wisniewski-Dyé F. Prigent-Combaret C. (2012). Which specificity in cooperation between phytostimulating rhizobacteria and plants? *Res. Microbiol.* 163 500–510. 10.1016/j.resmic.2012.08.006 22989671

[B35] Dutta S. Podile A. R. (2010). Plant growth promoting rhizobacteria (PGPR): the bugs to debug the root zone. *Crit. Rev. Microbiol.* 36 232 10.3109/10408411003766806 20635858

[B36] Dyachok J. Wiweger M. Kenne L. von Arnold S. (2002). Endogenous nod-factor-like signal molecules promote early somatic embryo development in Norway spruce. *Plant Physiol.* 128 523–533. 10.1104/pp.010547 11842156 PMC148915

[B37] East R. (2013). Soil science comes to life: Plants may be getting a little help with their tolerance of drought and heat. *Nature* 501 18–19. 10.1038/501S18a 24005397

[B38] Elmore J. M. Liu J. Smith B. Phinney B. Coaker G. (2012). Quantitative proteomics reveals dynamic changes in the plasma membrane during *Arabidopsis* immune signaling. *Mol. Cell. Proteom.* 14 1796–1813. 10.1074/mcp.M111.014555 PMC3322570 22215637

[B39] Engelmoer D. J. P. Behm J. E. Kiers E. T. (2014). Intense competition between arbuscular mycorrhizal mutualists in an in vitro root microbiome negatively affects total fungal abundance. *Mol. Ecol.* 23 1584–1593. 10.1111/mec.12451 24050702

[B40] Estévez J. Soria Díaz M. E. Fernández de Córdoba F. Móron B. Manyan H. Gil A. (2009). Different andnew Nod factors produced by *Rhizobium tropici* CIAT899 following Na stress. *FEMS Microbiol. Lett.* 293 220–231. 10.1111/j.1574-6968.2009.01540.x 19260963

[B41] Evangelisti E. Rey T. Schornack S. (2014). Cross-interference of plant development and plant–microbe interactions. *Curr. Opin. Plant Biol.* 20 118–126. 10.1016/j.pbi.2014.05.014 24922556

[B42] Gaiero J. R. Mccall C. A. Thompson K. A. Day N. J. Best A. S. Dunfield K. E. (2013). Inside the root microbiome: bacterial root endophytes and plant growth promotion. *Am. J. Bot.* 100 1738–1750. 10.3732/ajb.1200572 23935113

[B43] Giles E. D. Oldroyd J. D. Murray P. S. P. Downie J. A. (2011). The rules of engagement in the legume-rhizobial symbiosis. *Annu. Rev. Genet.* 45 119–144. 10.1146/annurev-genet-110410-132549 21838550

[B44] Gough C. Cullimore J. (2011). Lipo-chitooligosaccharide signaling in endosymbiotic plant-microbe interactions. *Mol. Plant Microb. Interact.* 8 867–878. 10.1094/MPMI-01-11-0019 21469937

[B45] Gray E. J. Smith D. L. (2005). Intracellular and extracellular PGPR: commonalities and distinctions in the plant-bacterium signaling processes. *Soil Biol. Biochem.* 37 395–412. 10.1016/j.soilbio.2004.08.030

[B46] Guasch-Vidal B. Estévez J. Dardanelli M. S. Soria-Díaz M. E. de Córdoba F. F. Balog C. I. (2013). High NaCl concentrations induce the nod genes of *Rhizobium tropici* CIAT899 in the absence of flavonoid inducers. *Mol. Plant Microbe Interact.* 26 451–460. 10.1094/MPMI-09-12-0213-R 23216086

[B47] Hartmann A. Schikora A. (2012). Quorum sensing of bacteria and trans-kingdom interactions of N-acyl homoserine lactones with eukaryotes. *J. Chem. Ecol.* 38 704–713. 10.1007/s10886-012-0141-7 22648507

[B48] Hartmann A. Rothballer M. Hense B. A. Schröder P. (2014). Bacterial quorum sensing compounds are important modulators of microbe-plant interactions. *Front. Plant Sci.* 5:131 10.3389/fpls.2014.00131 PMC3986513 24782873

[B49] Hassan S. Mathesius U. (2012). The role of flavonoids in root–rhizosphere signalling: opportunities and challenges for improving plant–microbe interactions. *J. Exp. Bot.* 9 3429–3444. 10.1093/jxb/err430 22213816

[B50] He Z. Piceno Y. Deng Y. Xu M. Lu Z. DeSantis T. (2012). The phylogenic composition and structure of soil microbial communities shifts in response to elevated carbon dioxide. *Int. Soc. Microb. Ecol.* 6 259–272. 10.1038/ismej.2011.99 PMC3260516 21796217

[B51] Hirsch P. R. Mauchline T. H. (2012). Who’s who in the plant root microbiome? *Nat. Biotechnol.* 30 961–962. 10.1038/nbt.2387 23051815

[B52] Imhoff M. L. Bounoua L. Ricketts T. Loucks C. Harriss R. Lawrence W. T. (2004). Global patterns in human consumption of net primary production. *Nature* 429 870–873. 10.1038/nature02619 15215863

[B53] James E. K. (2000). Nitrogen fixation in endophytic and associative symbiosis. *Field Crops Res.* 65 197–209. 10.1016/S0378-4290(99)00087-8

[B54] Jung W. J. Mabood F. Souleimanov A. Park R. D. Smith D. L. (2008a). Chitinases produced by *Paenibacillus illinoisensis* and *Bacillus thuringensis* subsp. pakistani degrade Nod factor from *Bradyrhizobium japonicum*. *Microbiol. Res.* 163 345–349. 10.1016/j.micres.2006.06.013 16904303

[B55] Jung W. J. Mabood F. Souleimanov A. Smith D. L. (2008b). Effect of chitin hexamer and Thuricin 17 on lignification related and antioxidative enzymes of soybean plants. *J. Plant. Biol.* 51 145–149. 10.1007/BF03030724

[B56] Jung W. J. Mabood F. Souleimanov A. Smith D. L. (2011). Induction of defense-related enzymes in soybean leaves by class IId bacteriocins (thuricin 17 and bacthuricin F4) purified from Bacillus strains. *Microbiol. Res.* 167 14–19. 10.1016/j.micres.2011.02.004 21501957

[B57] Kembel S. W. O’Connor T. K. Arnold H. K. Hubbell S. P. Wright S. J. Green J. L. (2014). Relationships between phyllosphere bacterial communities and plant functional traits in a neotropical forest. *Proc. Natl. Acad. Sci. U.S.A.* 38 13715–13720. 10.1073/pnas.1216057111 25225376 PMC4183302

[B58] Kennedy I. R. Choudhury A. I. M. A. Kecske M. L. (2004). Non-symbiotic bacterial diazotrophs in crop-farming systems: can their potential for plant growth promotion be better exploited. *Soil Biol. Biochem.* 36 1229–1244. 10.1016/j.soilbio.2004.04.006

[B59] Khan W. Prithiviraj B. Smith D. L. (2008). Nod factor [Nod Bj V (C18: 1, MeFuc)] and lumichrome enhance photosynthesis and growth of corn and soybean. *J. Plant Physiol.* 165 1342–1351. 10.1016/j.jplph.2007.11.001 18190997

[B60] Kloepper J. W. Ryu C. M. Zhang S. (2004). Induced systemic resistance and promotion of plant growth by *Bacillus* spp. *Phytopathology* 94 1259–1266. 10.1094/PHYTO.2004.94.11.1259 18944464

[B61] Knack J. J. Wilcox L. W. Ané J.-M. Piotrowski M. J. Cook M. E. Graham J. M. (2015). Microbiomes of streptophyte algae and bryophytes suggest that a functional suite of microbiota fostered plant colonization of land. *Int. J. Plant Sci.* 176 405–420. 10.1086/681161

[B62] Koenig J. E. Spor A. Scalfone N. Fricker A. D. Stombaugh J. Knight R. (2011). Succession of microbial consortia in the developing infant gut microbiome. *Proc. Natl. Acad. Sci. U.S.A.* 108 4578–4585. 10.1073/pnas.1000081107 20668239 PMC3063592

[B63] Kuzyakov Y. Domanski G. (2000). Carbon input by plants into the soil. *Rev. J. Plant Nutr. Soil Sci.* 163 421–431. 10.1002/1522-2624(200008)163:4<421::AID-JPLN421>3.0.CO;2-R

[B64] Lakshmanan V. Kitto S. L. Caplan J. L. Hsueh Y.-H. Kearns D. B. Wu Y.-S. (2012). Microbe-associated molecular patterns-triggered root responses mediate beneficial rhizobacterial recruitment in *Arabidopsis*. *Plant Physiol.* 160 1642–1661. 10.1104/pp.112.200386 22972705 PMC3486800

[B65] Lebeis S. L. (2015). Greater than the sum of their parts: characterizing plant microbiomes at the community level. *Curr. Opin. Plant Biol.* 24 82–86. 10.1016/j.pbi.2015.02.004 25710740

[B66] Lee K. D. Gray E. J. Mabood F. Jung W. J. Charles T. Clark S. R. D. (2009). The class IId bacteriocin thuricin 17 increases plant growth. *Planta* 229 747–755. 10.1007/s00425-008-0870-6 19083012

[B67] Loiret F. G. Ortega E. Kleiner D. Ortega-Rode’s P. Rode’s R. Dong Z. (2004). A putative new endophytic nitrogen-fixing bacterium *Pantoea* sp. from sugarcane. *J. Appl. Microbiol.* 97 504 10.1111/j.1365-2672.2004.02329.x 15281930

[B68] Lugtenberg B. Kamilova F. (2009). Plant-growth-promoting rhizobacteria. *Annu. Rev. Microbiol.* 63 541–556. 10.1146/annurev.micro.62.081307.162918 19575558

[B69] Lundberg D. S. Lebeis S. L. Paredes S. H. Yourstone S. Gehring J. Malfatti S. (2012). Defining the core *Arabidopsis thaliana* root. *Nature* 488 86–90. 10.1038/nature11237 22859206 PMC4074413

[B70] Luo Y. Hui D. Zhang D. (2006). Elevated CO_2_ stimulates net accumulations of carbon and nitrogen in land ecosystems: a meta-analysis. *Ecology* 87 53–63. 10.1890/04-1724 16634296

[B71] Lv D. Ma A. Tang X. Bai Z. Qi H. Zhuang G. (2013). Profile of the culturable microbiome capable of producing acyl-homoserine lactone in the tobacco phyllosphere. *J. Environ. Sci.* 25 357–366. 10.1016/S1001-0742(12)60027-8 23596957

[B72] Mabood F. Gray E. J. Lee K. D. Supanjani S. Smith D. L. (2006a). Exploiting inter-organismal chemical communication for improved inoculants. *Can. J. Plant Sci.* 86 951–966. 10.4141/P05-102

[B73] Mabood F. Zhou X. Lee K. D. Smith D. L. (2006b). Methyl jasmonate, alone or in combination with genistein, and *Bradyrhizobium japonicum* increases soybean (*Glycine max* L.) plant dry matter production and grain yield under short season conditions. *Field Crops Res.* 95 412–419. 10.1016/j.fcr.2005.04.013

[B74] Mabood F. Zhou X. Smith D. L. (2014). Microbial signaling and plant growth promotion. *Can. J. Plant Sci.* 94 1051–1063. 10.4141/cjps2013-148

[B75] Marasco R. Rolli E. Ettoumi B. Vigani G. Mapelli F. Borin S. (2012). A drought resistance-promoting microbiome is selected by root system under desert farming. *PLoS ONE* 7:e48479 10.1371/journal.pone.0048479 PMC3485337 23119032

[B76] Masciarelli O. Llanes A. Luna V. (2014). A new PGPR co-inoculated with *Bradyrhizobium japonicum* enhances soybean nodulation. *Microbiol. Res.* 169 609–615. 10.1016/j.micres.2013.10.001 24280513

[B77] Mengual C. Schoebitz M. Azcón R. Roldán A. (2014). Microbial inoculants and organic amendment improves plant establishment and soil rehabilitation under semiarid conditions. *J. Environ. Manag.* 134 1–7. 10.1016/j.jenvman.2014.01.008 24463051

[B78] Nelson L. M. (2004). Plant growth promoting rhizobacteria (PGPR): prospects for new inoculants. *Crop Manag.* 3 10.1094/CM-2004-0301-05-RV

[B79] Nguyen T. H. N. Brechenmacher L. Aldrich J. T. Clauss T. R. Gritsenko M. A. Hixton K. K. (2012). Quantitative phosphoproeomic analysis of soybean root hairs inoculated with *Bradyrhisobium japonicum*. *Mol. Cell. Proteom.* 11 1140–1155. 10.1074/mcp.M112.018028 PMC3494206 22843990

[B80] Oláh B. Brière C. Bécard G. Dénarié J. Gough C. (2005). Nod factors and a diffusible factor from arbuscular mycorrhizal fungi stimulate lateral root formation in *Medicago truncatula* via the DMI1/DMI2 signalling pathway. *Plant J.* 44 195–207. 10.1111/j.1365-313X.2005.02522.x 16212600

[B81] Oldroyd G. E. D. (2013). Speak, friend, and enter: signalling systems that promote beneficial symbiotic associations in plants. *Nat. Rev. Microbiol.* 11 252–263. 10.1038/nrmicro2990 23493145

[B82] Oldroyd G. E. Murray J. D. Poole P. S. Downie J. A. (2010). The rules of engagement in the legume5 rhizobial symbiosis. *Annu. Rev. Genet.* 45 119–144. 10.1146/annurev-genet-110410-132549 21838550

[B83] Orrell P. Bennett A. E. (2013). How can we exploit above-belowground interactions to assist in addressing the challenges of food security? *Front. Plant Sci.* 4:432 10.3389/fpls.2013.00432 PMC3812866 24198821

[B84] Peiffer J. A. Spor A. Koren O. Jin Z. Tring S. G. Dangl J. L. (2013). Diversity and heritability of the maize rhizosphere microbiome under field conditions. *Proc. Natl. Acad. Sci. U.S.A.* 16 6548–6553. 10.1073/pnas.1302837110 23576752 PMC3631645

[B85] Pisa G. Magnani G. S. Weber H. Souza E. M. Faoro H. Monteiro R. A. (2011). Diversity of 16S rRNA genes from bacteria of sugarcane rhizosphere soil. *Braz. J. Med. Biol. Res.* 44 1215–1221. 10.1590/S0100-879X2011007500148 22042267

[B86] Porcel R. Aroca R. Ruiz-Lozano J. M. (2012). Salinity stress alleviation using arbuscular mycorrhizal fungi. A review. *Agron. Sustain. Dev.* 32 181–200. 10.1007/s13593-011-0029-x

[B87] Porras-Alfaro A. Bayman P. (2011). Hidden fungi, emergent properties: endophytes and microbiomes. *Annu. Rev. Phytopathol.* 49 291–315. 10.1146/annurev-phyto-080508-081831 19400639

[B88] Prithiviraj B. Zhou X. Souleimanov A. Khan W. K. Smith D. L. (2003). A host specific bacteria-to-plant signal molecule (Nod factor) enhances germination and early growth of diverse crop plants. *Planta* 216 437–445. 12520335 10.1007/s00425-002-0928-9

[B89] Prudent M. Salon C. Souleimanov A. Emery R. J. N. Smith D. L. (2015). Soybean is less impacted by water stress using *Bradyrhizobium japonicum* and thuricin-17 from *Bacillus thuringiensis*. *Agron. Sustain. Dev.* 35 749–757. 10.1007/s13593-014-0256-z

[B90] Quecine M. C. Araújo W. L. Rossetto P. B. Ferreira A. Tsui S. Lacava P. T. (2012). Sugarcane growth promotion by the endophytic bacterium *Pantoea agglomerans* 33.1. *Appl. Environ. Microbiol.* 78 7511–7518. 10.1128/AEM.00836-12 22865062 PMC3485700

[B91] Ragauskas A. J. Williams C. K. Davison B. H. Britovsek G. Cairney J. Eckert C. A. (2006). The path forward for biofuels and biomaterials. *Science* 311 484–489. 10.1126/science.1114736 16439654

[B92] Rastogi G. Coaker G. L. Leveau J. H. J. (2013). New insights into the structure and function of phyllosphere microbiota through high-throughput molecular approaches. *FEMS Microbiol. Lett.* 348 1–10. 10.1111/1574-6968.12225 23895412

[B93] Rastogi G. Sbodio A. Tech J. J. Suslow T. V. Coaker G. L. Leveau J. H. J. (2012). Leaf microbiota in an agroecosystem: spatiotemporal variation in bacterial community composition on field-grown lettuce. *ISME J.* 6 1812–1822. 10.1038/ismej.2012.32 22534606 PMC3446804

[B94] Rose C. M. Venkateshwaren M. Volkening J. D. Grimsrud P. A. Maeda J. Bailey D. J. (2012). Rapid phosphoproteomic and transcriptomic changes in the rhizobia-legume symbiosis. *Mol. Cell. Proteom.* 11 724–744. 10.1074/mcp.M112.019208 PMC3434772 22683509

[B95] Rout M. E. Southworth D. (2013). The root microbiome influences scales from molecules to ecosystems: the unseen majority. *Am. J. Bot.* 100 1689–1691. 10.3732/ajb.1300291 24008514

[B96] Rudrappa T. Czymmek K. J. Paré P. W. Bais H. P. (2008). Root-secreted malic acid recruits beneficial soil bacteria. *Plant Physiol.* 148 1547–1556. 10.1104/pp.108.127613 18820082 PMC2577262

[B97] Ruiz-Lozano J. M. Porcel R. Azcon C. Aroca R. (2012). Regulation by arbuscular mycorrhizae of the integrated physiological response to salinity in plants: new challenges in physiological and molecular studies. *J. Exp. Bot.* 11 4033–4044. 10.1093/jxb/ers126 22553287

[B98] Santi C. Bogusz D. Franche C. (2013). Biological nitrogen fixation in non-legume plants. *Ann. Bot.* 111 743–767. 10.1093/aob/mct048 23478942 PMC3631332

[B99] Schmidt R. Köberl M. Mostafa A. Ramadan E. M. Monschein M. Jensen K. B. (2014). Effects of bacterial inoculants on the indigenous microbiome and secondary metabolites of chamomile plants. *Front. Microbiol.* 64:111 10.3389/fmicb.2014.00064 PMC3928675 24600444

[B100] Schwinghamer T. Souleimanov A. Dutilleul P. Smith D. L. (2014). The plant growth regulator lipo-chitooligosaccharide (LCO) can enhance the germination of canola (*Brassica napus* [L.]*)*. *J. Plant Growth Regul.* 34 183–195. 10.1007/s00344-014-9456-7

[B101] Segonzac C. Zipfel C. (2011). Activation of plant pattern-recognition receptors by bacteria. *Curr. Opin. Microbiol.* 14 54–61. 10.1016/j.mib.2010.12.005 21215683

[B102] Smith D. L. (2009). “Signals in the underground: microbial signals and plant productivity,” in *Proceedings of the International Society for Molecular Plant Microbe Interactions Meeting, July* 19–23, Quebec City.

[B103] Smith D. L. (2010). “Signals coming in from the cold: inter-organismal communication and abiotic stress,” in *Proceedings of the the 21st North American Symbiotic Nitrogen Fixation Conference* (Columbia, MI: University of California Energy Week).

[B104] Smith D. L. Zhou X. (2014). An effective integrated research approach to study climate change in Canada. *Can. J. Plant. Sci.* 94 995–1008. 10.4141/cjps-2014-503

[B105] Souleimanov A. Prithiviraj B. Smith D. L. (2002). The major Nod factor of Bradyrhizobium japonicum promotes early growth of soybean and corn. *J. Exp. Bot.* 53 1929–1934. 10.1093/jxb/erf034 12177132

[B106] Spence C. Alff E. Johnson C. Ramos C. Donofrio N. Sundarsan V. (2014). Natural rice rhizospheric microbes suppress rice blast infections. *BMC Plant Biol.* 14:130 10.1186/1471-2229-14-130 PMC4036093 24884531

[B107] Subramanian S. (2014). *Mass Spectrometry Based Proteome Profiling to Understand the Effects of Lipo-Chitooligosaccharide and Thuricin 17 in Arabidopsis thaliana and Glycine max under Salt Stress.* Ph.D. Thesis, McGill University, Montréal, QC.

[B108] Subramanian S. Mitkus E. Souleimanov A. Smith D. L. (2011). “Thuricin 17 and lipo-chito oligosaccharide act as plant growth promoters and alleviate drought stress in *Arabidopsis thaliana,*” in *Proceedings of the Eastern Regional Meeting of the Canadian Society of Plant Physiologists* (Ottawa: Carleton University).

[B109] Subramanian S. Schwinghamer T. Souleimanov A. Smith D. L. (2009). “Evaluating thuricin 17 (TH17) and lipo-chito oligosaccharide (LCO) as plant growth promoter in *Arabidopsis thaliana*,” in *Proceedings of the MPMI International Meeting*, Quebec City.

[B110] Subramanian S. Souleimanov A. Smith D. L. (2010). “Thuricin 17 and lipo-chitooligosaccharide act as plant growth promoters and alleviate low temperature stress in *Arabidopsis thaliana*,” in *Proceedings of the North American Symbiotic Nitrogen Fixation Conference, June 14-17, 010* Columbia, MI.

[B111] Taylor T. N. (1995). Fossil arbuscular mycorrhizae from the Early Devonian. *Mycologia* 87 560–573. 10.1098/rsbl.2010.1203

[B112] Tena G. Boudsocq M. Sheen J. (2011). Protein kinase signaling networks in plant innate immunity. *Curr. Opin. Plant Biol.* 14 519–529. 10.1016/j.pbi.2011.05.006 21704551 PMC3191242

[B113] Teplitski M. Robinson J. B. Bauer W. D. (2000). Plants secrete substances that mimic bacterial N-Acyl homoserine lactone signal activities and affect population density-dependent behaviors in associated bacteria. *Mol. Plant Microbe Interact.* 6 637–648. 10.1094/MPMI.2000.13.6.637 10830263

[B114] Trabelsi D. Mhamdi R. (2013). Microbial inoculants and their impact on soil microbial communities: a review. *Biomed. Res. Int.* 1 13 10.1155/2013/863240 PMC3728534 23957006

[B115] Turner T. R. James E. K. Poole P. S. (2013a). The plant microbiome. *Genome Biol.* 14 209–219. 10.1186/gb-2013-14-6-209 23805896 PMC3706808

[B116] Turner T. R. Ramakrishnan K. Walshaw J. Heavens D. Alston M. Swarbreck D. (2013b). Comparative metatranscriptomics reveals kingdom level changes in the rhizosphere microbiome of plants. *ISME J.* 7 2248–2258. 10.1038/ismej.2013.119 23864127 PMC3834852

[B117] Velázquez E. Rojas M. Lorite M. J. Rivas R. Zurdo-Piñeiro J. L. Heydrich M. (2008). Genetic diversity of endophytic bacteria which could be find in the apoplastic sap of the medullary parenchyma of the stem of healthy sugarcane plants. *J. Basic Microbiol.* 48 118–124. 10.1002/jobm.200700161 18383223

[B118] Venkateshwaran M. Volkening J. D. Sussman M. R. Ané J.-M. (2013). Symbiosis and the social network of higher plants. *Curr. Opin. Plant Biol.* 16 118–127. 10.1016/j.pbi.2012.11.007 23246268

[B119] Vessey J. K. (2003). Plant growth promoting rhizobacteria as biofertilizers. *Plant Soil* 255 571–586. 10.1023/A:1026037216893

[B120] Von Bulow J. F. W. Dobereiner J. (1975). Potential for nitrogen fixation in maize genotypes in Brazil. *Proc. Natl. Acad. Sci. U.S.A.* 72 2389–2393. 10.1073/pnas.72.6.2389 16592251 PMC432764

[B121] Wang N. Khan W. Smith D. L. (2012). Soybean global gene expression after application of lipo-chitooligosaccharide from *Bradyrhizobium japonicum* under sub-optimal temperature. *PLoS ONE* 7:e31571 10.1371/journal.pone.0031571 PMC3278468 22348109

[B122] Watrous J. Roach P. Alexandrov T. Heath B. S. Yang J. Y. Kersten R. D. (2012). Mass spectral molecular networking of living microbial colonies. *Proc. Natl. Acad. Sci. U.S.A.* 109 E1743–E1752. 10.1073/pnas.1203689109 22586093 PMC3387089

[B123] Wittulsky S. Pellegrin C. Giannakopoulou A. Böni R. (2014). A snapshot of molecular plant-microbe interaction research. *New Phytol.* 205 468–471. 10.1111/nph.13194 25521069

[B124] Zamioudis C. Mastranesti P. Dhonukshe P. Blilou I. Pieterse C. M. J. (2013). Unraveling root developmental programs initiated by beneficial pseudomonas spp. Bacteria. *Plant Physiol.* 162 304–318. 10.1104/pp.112.212597 23542149 PMC3641211

[B125] Zhang F. Smith D. L. (1995). Preincubation of Bradyrhizobium japonicum with genistein accelerates nodule development of soybean at suboptimal root zone temperatures. *Plant Phys.* 108 961–986. 10.1104/pp.108.3.961 PMC157445 12228519

[B126] Zhang F. Smith D. L. (2002). Interorganismal signaling in suboptimal environments: The legume-rhizobia symbiosis. *Adv. Agron.* 76 125–161. 10.1016/S0065-2113(02)76004-5

[B127] Zhang H. Gao Z.-Q. Wang W.-J. Liu G.-F. Shtykova E. V. Xu J.-H. (2012). The crystal structure of the MPN domain from the COP9 signalosome subunit CSN6. *FEBS Lett.* 586 1147–1153. 10.1016/j.febslet.2012.03.029 22575649

[B128] Zolla G. Badri D. V. Bakker M. G. Manter D. K. Vivanco J. M. (2013). Soil microbiomes vary in their ability to confer drought tolerance to *Arabidopsis*. *Appl. Soil Ecol.* 68 1–9. 10.1016/j.apsoil.2013.03.007

